# Ultrasonographic Evaluation of Peripheral Nerves for Early Diagnosis of Guillain–Barré Syndrome: A Case‐Control Study

**DOI:** 10.1002/hsr2.71655

**Published:** 2025-12-14

**Authors:** Gholamreza Shamsaei, Mitra Sadrian, Amir Hossein Karimi, Shooka Mohammadi

**Affiliations:** ^1^ Department of Neurology, School of Medicine Ahvaz Jundishapur University of Medical Sciences Ahvaz Iran

**Keywords:** electrophysiology, Guillain–Barré syndrome, median nerve, nerve conduction studies, peripheral nerves, ulnar nerve, ultrasonography

## Abstract

**Background and Aim:**

Guillain‐Barré syndrome (GBS) is an acute immune‐mediated neuropathy in which early diagnosis remains challenging because nerve conduction studies (NCS) may appear normal in the first few days after onset. Ultrasonography (US) has recently gained attention as a non‐invasive tool for evaluating peripheral nerve pathology. This study aimed to assess the usefulness of ultrasonographic cross‐sectional area (CSA) measurement of peripheral nerves for the early diagnosis of GBS in Iranian patients within 2 weeks of GBS symptom onset.

**Methods:**

In this case‐control study, 150 participants were enrolled, including 75 patients with GBS and 75 healthy controls matched for age and sex. Patients were referred to Golestan Hospital (Ahvaz, Iran) between November 2023 and June 2025 with less than 2 weeks of GBS onset. All participants underwent demographic and clinical assessments, NCS, and US evaluations of the ulnar and median nerves.

**Results:**

Each group of participants (patients or controls) comprised 47 men and 28 women. The mean age of the patients with GBS was 43.8 ± 10.9 years. Statistically significant mean differences were found in all measured variables of the ulnar and median nerves, including ultrasonographic CSAs and NCS parameters (F‐wave latency and persistence, motor nerve conduction velocity (MCV), sensory nerve conduction velocity (SCV), sensory nerve action potentials (SNAP), distal motor latency (DML), and compound muscle action potential (CMAP)), between patients with GBS and those in the control group (*p* < 0.05). The mean ultrasonographic CSAs of the ulnar and median nerves were significantly larger in patients with GBS than in the control group (*p* < 0.05).

**Conclusion:**

Sonographic measurement of the CSA of the ulnar and median nerves differentiates early‐stage GBS patients from healthy controls and may support early diagnosis. The combination of US and NCS provides complementary information that facilitates early recognition of GBS.

## 1 Introduction

Guillain‐Barré syndrome (GBS) is an acute autoimmune disease characterized by the rapid onset of paralysis and muscle weakness, often following an infectious episode [1, 2, 3, 4, 5, 6, 7]. The disease is caused by antibodies targeting the myelin sheath surrounding spinal nerve roots [8, 9, 10]. Initial symptoms typically appear within days to a week after infection and generally progress over 2 weeks, with more than 90% of patients developing severe disease within 4 weeks [11]. GBS encompasses multiple subtypes, each with distinct pathophysiological mechanisms and clinical presentations [1, 2, 7].

The diagnosis of GBS is primarily clinical and is supported by laboratory tests and nerve conduction studies (NCS) [12, 13]. Electrophysiological testing can differentiate between demyelinating and axonal subtypes and provide prognostic information [14]. However, NCS may yield normal results in the first week of the disease or in cases of mild proximal weakness, limiting its utility for early diagnosis [15].

Peripheral nerve ultrasonography (US) has recently gained attention as a non‐invasive, cost‐efficient tool for evaluating structural nerve changes in various neuropathies [16, 17, 18, 19, 20]. While NCS evaluates nerve function, US provides complementary information about nerve morphology, allowing the identification of enlargement or swelling that correlates with disease severity and subtype in GBS [21, 22, 23, 24, 25, 26, 27]. Serial US evaluations can also be used to monitor disease progression and treatment responses, offering additional prognostic insights [28]. The combination of US and NCS has been reported to improve the overall assessment of peripheral nerves [21].

Few studies have assessed the role of US in the early diagnosis of GBS [23, 25, 29, 30], and data from the Middle East, particularly Iran, on the utility of US for early GBS detection are limited. Electrophysiological tests, the current standard for diagnosis, often show reduced sensitivity during the first 2 weeks of GBS symptom onset, which can delay timely intervention. US is capable of visualizing peripheral nerve structures and may serve as a valuable adjunct for early diagnosis. Therefore, this study aimed to assess the diagnostic value of peripheral nerve US for early detection of GBS in Iranian patients within the first 2 weeks of symptom onset.

## 2 Materials and Methods

### 2.1 Study Design and Participants

This case–control study was performed among Iranian patients with GBS who were referred to the Neurology Clinic of Golestan Hospital (Ahvaz, Iran) between November 2023 and June 2025. An equal number of age‐ and sex‐matched healthy controls were recruited. Ethical approval was obtained from the Ethics Committee of Ahvaz Jundishapur University of Medical Sciences (NO: IR.AJUMS.HGOLESTAN.REC.1402.178), and written informed consent was obtained from all participants. All eligible patients with GBS and controls were enrolled according to the predefined inclusion and exclusion criteria.

### 2.2 Inclusion and Exclusion Criteria

GBS was diagnosed according to the Brighton criteria (levels 1–3) [11] based on clinical presentation and initial electrophysiological findings. Eligible cases were adults (≥ 18 years) presenting with acute, symmetric weakness of the lower and upper limbs, reduced or absent deep tendon reflexes, and symptom progression over several days, up to a maximum of 2 weeks. The controls were healthy adults without neurological disorders or systemic comorbidities.

The exclusion criteria were spinal or cranial cord lesions, hereditary neuropathies, medication‐induced neuropathy, systemic diseases affecting peripheral nerves (e.g., chronic liver disease, uremia, diabetes mellitus), a history of GBS or chronic inflammatory demyelinating polyneuropathy (CIDP), symptom onset more than 2 weeks before evaluation, and refusal to undergo US assessment. Patients requiring mechanical ventilation or intensive care were also excluded.

### 2.3 Clinical and Laboratory Evaluation

All patients with GBS and controls underwent NCS and US assessments of their median and ulnar nerves. Demographic and clinical information were recorded using a standardized checklist. Lumbar puncture was performed when indicated to assess cerebrospinal fluid (CSF) characteristics. A CSF protein concentration > 60 mg/dL was considered supportive of GBS [11]. Muscle strength was evaluated using the Medical Research Council Sum Score (MRCSS), which ranges from 0 (complete paralysis) to 60 (normal strength). Disability was assessed using the Hughes Disability Scale (HDS), with scores ranging from 0 (normal) to 6 (death).

### 2.4 Electrophysiological Studies

A neurologist with expertise in electrodiagnostic testing, blinded to participants' clinical status, performed all NCS in accordance with established guidelines [31]. Examinations were performed with the participants in the supine position, and the skin temperature was maintained above 32°C. The median and ulnar nerves were evaluated for F‐wave latency and persistence, sensory nerve conduction velocity (SCV), compound muscle action potential (CMAP), sensory nerve action potentials (SNAP), motor nerve conduction velocity (MCV), and distal motor latency (DML). Supramaximal percutaneous stimulation with a constant‐current stimulator and surface electrodes was used in all studies. Electrodiagnostic classification followed the established criteria [31].

### 2.5 Ultrasonography Evaluation

Ultrasonography of the ulnar and median nerves was performed by an experienced neurologist using a Sonosite M‐Turbo system equipped with a high‐frequency linear probe (5–17 MHz, routinely operated at 10–13 MHz). The clinical and electrophysiological findings were kept confidential from the sonographer, and all imaging was conducted by a single examiner to ensure result consistency.

The participants were evaluated in the supine position. B‐mode imaging was used to visualize each nerve with a scanning depth of 3 cm and an individually adjusted focal point. The transducer was applied with minimal pressure to avoid nerve deformation and positioned perpendicular to the nerve to obtain the most accurate and smallest cross‐sectional area (CSA). Color Doppler imaging was used to distinguish adjacent vascular structures from the nerves.

For each nerve, the CSA was measured by manually tracing the inner border of the hyperechoic epineurium using the built‐in ellipse‐tracing tool of the ultrasonography device. Two consecutive measurements were obtained at each anatomical site, and the average of the two values was used for analysis. The CSAs of the median nerve were assessed at two anatomical sites: the distal forearm (≥ 5 cm proximal to the carpal tunnel) and the proximal arm near the elbow. The ulnar nerve was assessed at the mid‐arm (proximal) and mid‐forearm (distal) regions, in accordance with established protocols [23, 32, 33].

### 2.6 Statistical Analysis

Statistical analyses were performed using SPSS version 24 (IBM Corp., Armonk, NY, USA). The Shapiro–Wilk test was used to assess data normality. Continuous variables were presented as mean ± standard deviation (SD), and categorical variables as frequencies and percentages. Independent‐samples *t*‐test was used for normally distributed continuous variables, and a chi‐square test was applied for the categorical data. Statistical significance was defined as a p‐value less than 0.05.

## 3 Results

In this case‐control study, 150 participants were enrolled and evenly divided into two groups: 75 patients with GBS and 75 healthy controls. Both groups were matched for age and sex. The demographic and clinical characteristics of the participants are displayed in Table 1. Each group included 47 men and 28 women. The mean age of the patients was 43.8 ± 10.9 years (range, 23–60). A significant difference in mean BMI was observed between the two groups (*p* < 0.01).

**Table 1:** Demographic and clinical characteristics of GBS patients and the control group (*n* = 150).

Characteristics			Mean ± SD		*p* value
			Patients with GBS (*n* = 75)	Control group (*n* = 75)	
Age (years)			43.80 ± 10.88	43.96 ± 10.75	> 0.05
BMI (kg/m^2^)			26.89 ± 1.88	23.50 ± 1.50	< 0.05
MRCSS on admission			47.77 ± 3.21	—	—
CSF (mg/dL)			68.42 ± 4.23	—	—
			***n* (%)**		
Gender	Male		47 (62.7)	47 (62.7)	> 0.05
	Female		28 (37.3)	28 (37.3)	
Age group	20–30		33 (44)	33 (44)	> 0.05
	31–40		16 (21.3)	16 (21.3)	
	41–50		26 (34.7)	26 (34.7)	
BMI	18.5–24.9		10 (13.3)	75 (100)	< 0.05
	25–29.9		57 (76)	—	
	> 30		8 (10.7)	—	
Subtypes of GBS	AIDP		54 (72)	—	
	AMSAN		15 (20)	—	
	AMAN		6 (8)	—	
MRCSS	51–60		20 (26.7)	—	
	41–50		25 (33.7)	—	
	31–40		15 (20)	—	
	21–30		8 (10.7)	—	
	20≥		7 (9.3)	—	
HDS	1	Minor symptoms, able to run	11 (14.7)	—	—
	2	Able to walk ≥ 10 m without assistance, but unable to run	23 (30.7)	—	—
	3	Able to walk 10 meters with assistance	17 (22.7)	—	—
	4	Bedridden or chair‐bound	24 (32)	—	—

Abbreviations: AIDP, acute inflammatory demyelinating polyradiculoneuropathy; AMSAN, acute motor sensory axonal neuropathy; AMAN, acute motor axonal neuropathy; BMI, body mass index; CSF, cerebrospinal fluid; GBS, Guillain‐Barré syndrome; HDS, Hughes Disability Scores; MRCSS, Medical Research Council sum score; US, ultrasonography.

Based on the HDS on admission, 11 patients (14.7%) had mild symptoms and were able to run, while 41 (54.7%) had HDS ≥ 3, indicating that they required assistance to walk or were bedridden. The mean CSF protein concentration in the patients was 68.42 ± 4.23 mg/dL. In terms of subtype distribution, 21 patients had the axonal subtype and 54 had the demyelinating subtype of GBS. Regarding muscle strength, 26.7% and 33.7% of patients had MRCSS of 51–60 and 41–50, respectively, with a mean MRCSS of 47.77 ± 3.21 at admission.

A comparison of the ultrasonographic CSAs of the median and ulnar nerves between GBS patients (within 2 weeks of onset) and the control group is presented in Table 2. The mean CSAs of both nerves were significantly larger in patients with GBS than in controls at both proximal and distal sites (*p* < 0.05). Table 3 summarizes the electrophysiological findings of the median and ulnar nerves in GBS patients (within 2 weeks of onset) and the control group. Significant differences were observed between the GBS and control groups in all NCS parameters for both the ulnar and median nerves, including F‐wave latency and persistence, DML, MCV, SCV, CMAP, and SNAP (all *p* < 0.05).

**Table 2:** Comparison of ultrasonographic CSA of median and ulnar nerves in GBS patients (within 2 weeks of GBS onset) and the control group (*n* = 150).

	**Mean ± SD**		
Ultrasonography findings	Patients with GBS (*n* = 75)	Control group (*n* = 75)	*p* value
Median nerve			
CSA, proximal (mm^2^)	13.44 ± 2.81	9.63 ± 2.72	< 0.05
CSA, distal (mm^2^)	11.97 ± 3.17	8.35 ± 2.15	< 0.05
Ulnar nerve			
CSA, proximal (mm^2^)	9.82 ± 2.33	7.23 ± 1.75	< 0.05
CSA, distal (mm^2^)	9.23 ± 2.77	6.21 ± 1.83	< 0.05

Abbreviations: CSA, cross‐sectional area; GBS, Guillain–Barré.

**Table 3:** Comparison of electrophysiological findings of median and ulnar nerves in GBS patients (within two weeks of GBS onset) and the control group (*n* = 150).

		Mean ± SD		
Electrophysiological findings		Patients with GBS (*n* = 75)	Control group (*n *= 75)	*p* value
**Median nerve**	F‐wave latency (ms)	35.56 ± 12.65	27.56 ± 3.97	< 0.05
	F‐wave persistence **(**%)	45.23 ± 13.28	96.38 ± 9.47	< 0.05
	DML (ms)	5.49 ± 1.28	3.91 ± 0.81	< 0.05
	MCV (m/s)	46.97 ± 14.15	55.83 ± 5.31	< 0.05
	SCV (m/s)	43.17 ± 5.42	54.48 ± 3.44	< 0.05
	CMAP (mV)	6.21 ± 2.18	11.75 ± 4.64	< 0.05
	SNAP (µV)	22.61 ± 6.61	31.34 ± 7.53	< 0.05
**Ulnar nerve**	F‐wave latency (ms)	34.93 ± 13.28	26.56 ± 3.77	< 0.05
	F‐wave persistence (%)	49.51 ± 8.22	95.22 ± 11.34	< 0.05
	DML (ms)	4.46 ± 1.89	2.83 ± 0.41	< 0.05
	MCV (m/s)	45.51 ± 11.24	57.18 ± 6.13	< 0.05
	SCV (m/s)	46.44 ± 7.21	57.85 ± 5.35	< 0.05
	CMAP (mV)	5.65 ± 3.18	13.33 ± 5.11	< 0.05
	SNAP (µV)	23.92 ± 5.14	32.21 ± 5.16	< 0.05

Abbreviations: CMAP, compound muscle action potential; DML, distal motor latency; mV, millivolts; MCV, motor conduction velocity; ms, milliseconds; m/s, meters per second; SCV, sensory conduction velocity; SNAP, sensory nerve action potential; µV, microvolts.

## 4 Discussion

A total of 150 participants were enrolled in this case–control study, including 75 patients with GBS and 75 sex‐matched healthy controls (47 men and 28 women per group). The mean age of the patients was 43.8 ± 10.9 years (range, 23–60). Among the patients, 21 had the axonal subtype and 54 had the demyelinating subtype. These findings are consistent with previous studies from Egypt [25] and Iran [34], which also described a predominance of the demyelinating subtype and a higher male prevalence. Similar age distributions were reported in studies from Malaysia [35] and Iran [34], where most patients were aged 30‐50 years. The higher prevalence in men [36] may reflect greater exposure to infection and environmental pathogens [34].

At admission, 11 patients exhibited mild symptoms and were able to walk or run independently, while 41 had HDS ≥ 3, indicating dependence on assistance or wheelchair use. The mean CSF protein concentration was 68.42 ± 4.23 mg/dL. MRCSS of 51–60 and 41–50 was observed in 26.7% and 33.7% of patients, respectively (mean = 47.77 ± 3.21), reflecting moderate to marked weakness and highlighting functional variability at presentation. The US showed significantly larger CSAs in both the proximal and distal segments of the median and ulnar nerves in GBS patients compared to controls, indicating early structural nerve changes. Significant differences were also identified in NCS parameters, including F‐wave latency and persistence, DML, MCV, SCV, CMAP, and SNAP, consistent with previous findings [37].

A recent systematic review and meta‐analysis highlighted that peripheral nerve US provides valuable diagnostic insights into GBS and can facilitate early recognition and management of the disease [38]. A study from Malaysia using serial nerve US reported significant increases in the CSA of the ulnar and median nerves within the first 3 weeks of disease onset, highlighting the value of serial US in monitoring structural changes and gradual recovery in patients with GBS [35]. Another study using nerve US reported that all sensorimotor nerve CSAs were significantly larger in patients with GBS at disease onset than in healthy controls [25].

Prolonged F‐wave latency, particularly in the ulnar and median nerves, is one of the earliest electrophysiological abnormalities observed in patients with GBS [15, 39, 40]. Variability in CSA may aid in classifying GBS subtypes, thereby improving diagnostic precision and guiding management [24, 41]. The US can help differentiate demyelinating from axonal subtypes, even when early NCS results appear normal, as electrophysiological abnormalities often develop days later [39, 40].

Multiple studies have emphasized the complementary roles of US and NCS in the early diagnosis, subtype classification, and severity assessment of GBS [25, 37, 42, 43]. US enables direct visualization of structural changes, early pathological detection, and high reproducibility [44, 45, 46], allowing identification of inflammatory edema, demyelination, and blood–nerve barrier disruption before electrophysiological abnormalities become evident [23, 25, 35]. US of peripheral nerves has proven to be a reliable and reproducible diagnostic tool in clinical practice [20, 47, 48, 49, 50]. It may be more sensitive than electrophysiological testing in the early stages of GBS, detecting CSA enlargement in most affected nerves, even when electrophysiological studies show only subtle changes such as prolonged F‐wave latencies [23, 25]. These findings underscore the role of US as a valuable adjunct to NCS for early GBS diagnosis. Moreover, US can be particularly useful when NCS is unavailable or inconclusive during the initial phase of GBS, facilitating earlier initiation of immunotherapy and potentially improving patient outcomes.

In the present study, all patients exhibited abnormal NCS findings, likely because the evaluations were conducted within the first 2 weeks of symptom onset, when abnormalities are typically detectable. No patients with normal NCS but abnormal US findings were identified. Nevertheless, previous studies have suggested that nerve enlargement on US can precede detectable NCS changes, particularly within the first week of GBS [23, 35].

This study contributes to the limited research on the US evaluation of GBS in Iran. These findings demonstrate significantly increased CSAs of the ulnar and median nerves in patients with early‐stage GBS compared with healthy controls, confirming that US provides structural information complementary to NCS. However, US images should always be interpreted in conjunction with clinical and electrophysiological data for a comprehensive diagnosis. The median and ulnar nerves were selected because of their superficial course, ease of visualization, and well‐established normative CSA values. These nerves are readily accessible and provide reproducible measurements, often showing early electrophysiological and ultrasonographic alterations consistent with proximal demyelination in GBS. In contrast, imaging lower limb nerves was more difficult due to their deeper position, smaller size, and surrounding soft tissues, which can impact image quality.

The limitations of this study include its single‐center design, relatively small sample size, modest BMI imbalance, exclusion of mechanically ventilated patients, restriction to the median and ulnar nerves, and absence of repeatability data. Sensitivity, specificity, and Receiver Operating Characteristic (ROC) analyses were not feasible because of the case–control design. Subgroup analysis by GBS subtype was not performed due to the limited number of axonal cases, which restricted the statistical comparisons. Prior studies suggest that nerve enlargement is typically more pronounced in demyelinating versus axonal subtypes [24, 27], a finding that requires confirmation in larger patient cohorts. Future multicenter studies with larger, more diverse cohorts should include both upper and lower limb nerve assessments and perform early serial evaluations within the first week of illness. Longer follow‐up is also needed to validate these findings, establish diagnostic thresholds, and clarify CSA variations across GBS subtypes.

## 5 Conclusion

In early GBS, the CSA of the median and ulnar nerves measured by US is significantly increased compared to that in healthy controls, reflecting early structural changes in the nerves. US is a reliable and accessible adjunct to NCS, and the combined use of them enhances diagnostic accuracy and supports timely therapeutic interventions. Future large‐scale multicenter studies are warranted to validate these findings.

## Author Contributions

**Gholamreza Shamsaei:** project administration, conceptualization, investigation, funding acquisition, methodology, writing – review and editing, supervision. **Mitra Sadrian:** conceptualization, invesitgation, writing – review and editing, supervison. **Amir Hossein Karimi:** investigation, data curation, writing – review and editing. **Shooka Mohammadi:** software, formal analysis, writing – review and editing, writing – original draft.

## Funding

The authors received no specific funding for this work.

## Disclosure

The lead author Mitra Sadrian affirms that this manuscript is an honest, accurate, and transparent account of the study being reported; that no important aspects of the study have been omitted; and that any discrepancies from the study as planned (and, if relevant, registered) have been explained.

## Ethics Statement

Ethical approval was obtained from the Medical Ethics Committee of Ahvaz Jundishapur University of Medical Sciences (AJUMS) (NO: IR.AJUMS.HGOLESTAN.REC.1402.178).

## Conflicts of Interest

The authors declare no conflicts of interest.

## Data Availability

The data analyzed in this study can be obtained from the corresponding author upon request.
